# Voices in Evidence-Based Newborn Care: A How-to-Guide on Developing a Parent-Facing Podcast

**DOI:** 10.2196/16335

**Published:** 2019-12-20

**Authors:** Joanna Parga-Belinkie, Raina M Merchant

**Affiliations:** 1 Children's Hospital of Philadelphia Philadelphia, PA United States; 2 University of Pennsylvania Philadelphia, PA United States

**Keywords:** neonatology, social media, medical education, patient education

## Abstract

Podcasting is becoming a more popular form of media. Its use in medical education is being researched—but what about its use in public education? In this tutorial, the authors offer a how-to-guide on starting a public or patient-facing podcast. The authors hope to inspire more physicians to utilize this type of media to share evidence-based information. More research is needed looking into how podcasting can be used to help with patient education.

## Introduction

As a neonatologist and new parent, the birth of my daughter brought with it a search for the *right way* to raise my healthy newborn. Although well versed in infant pathology, I did not know the differences in pacifier brands, how to work a breast pump, or if there was an ergonomically superior baby carrier. Parenting took my pediatric training, mixed it with a chronic as opposed to episodic sleep deprivation, upset my understanding of practical knowledge in my field, and generally upended me. I know I am not alone in feeling this way. Being disoriented and distressed is common after delivery. Baby blues strike 80% of new parents [1]. If symptoms of sadness persist for 2 weeks, concerns for postpartum depression arise [2]. Postpartum depression affects an estimated 15% of new parents [2]. To ease my unrest, I searched for solid and easily available evidence-based parenting resources. Despite being educated about scientific studies and research, I found myself confused by infant care claims on the internet and social media.

I started to wonder how parents without a medical background were navigating the highly commercialized and often unevidenced world of modern parenting. After researching different forms of media, it became evident that podcasting was gaining a growing listenership [3]. For the first time in history, more than half of the Americans (51%) have reported listening to podcasts, that is, 144 million people up from approximately 1.4 million a decade ago in 2009 [3]. However, within the podcasting landscape there were no prominent female physician voices leading medical education for pregnancy. I saw this as an opportunity to create something in this space. I looked to starting a podcast as a way to humanize my experiences in parenting and provide factual information and commentary on infant development, practices, and products when applicable. To accomplish this, I enlisted the help of another neonatologist to cohost the episodes, and began utilizing resources within my academic institution to help with podcast growth. This study is about how to start a medical podcast, and it outlines the successes and pitfalls my team has experienced as physician podcasters.

### Reviewing the Parenting Podcast Landscape

Before starting a podcast, we looked at what was already available for parents regarding pregnancy and the first 3 to 4 months of an infant’s life [4,5]. Although podcasts existed on parenting experiences and breastfeeding, there were none we could find by physicians dedicated to the postpartum period. For a lot of the podcasts, the hosts were not experts in infant care and relied on recounting their personal experiences or bringing on experts for topic interviews. Physicians did host podcasts, but the majority were aimed at educating other clinicians as opposed to the public [6]. There was room for a podcast that allowed for medical providers to recount their clinical experiences to those in nonmedical professions. This would allow for the creation of a trusted voice for medical information that could be listened to outside of the doctor’s office.

### Preparing to Launch and Financial Considerations

Purveying medical information can be dry—a hosting format between 2 women who are both doctors and mothers was devised to keep topics conversational, avoid monotony, and give different perspectives from providers within the field. Use of a pseudonym, in this case Baby Doctor Mamas, was also chosen. This would allow for various neonatologists, obstetricians, or gynecologists to act as hosts on the podcast. The hope was that this would ensure longevity and marketability of the podcast.

Launching the podcast required some startup capital that we paid out of pocket (Table 1).

**Table 1:** Startup costs for launch of the podcast Baby Doctor Mamas.

Item	Annual costs	Supplies/other services considered
Equipment	Approximately US $2000-US $3000; one-time fee	Basic supplies: recorder, 2 professional microphones
Website design and logo creation	Free, services offered by another neonatologist	Use of 99designs company
Jingle for Introduction	US $46.55; Jamendo website, full rights to content	Use of freelance composers
Domain name	US $12.17; GoDaddy website	None
Website hosting	US $132.00; Wordpress website with Bluehost server space	Use of wordpress.com but ultimately used wordpress.org given better user rights
Podcast hosting	US $89.00; Blubrry hosting service	Use of Libsyn or soundcloud services
Audio editing	US $20-30 per hour	Self-editing with Audacity, freelance sound designer
Total startup costs	US $198.72+ ongoing editing costs	Not applicable

Startup equipment included a Tascam recorder and Rode microphones. The recording space was a spare guest bedroom in one of the host’s house, with pillow padding around the microphones to buffer sounds. For help with logo creation and website development [7], another neonatologist, Dr Juanita Lewis, volunteered her services. Website development required purchase of a domain name and server space. A platform for hosting the podcast was also required, and Blubrry [8] was chosen for cost and statistical capabilities.

We wanted the podcast to sound professional as audio quality is an important metric for listener engagement. Instead of purchasing equipment for audio editing, we hired an audio editor who charged an hourly fee for edits. When launching, we decided on a regular schedule of once weekly release of the podcast to allow for predictability and growth—that way our listeners knew when to expect episode releases to follow them. We also had 4 episodes prerecorded before our launch, releasing 2 at once and allowing the other 2 to be released in the coming weeks. This allowed for varied content at the outset of the podcast, for effective time management, and for us to be prepared for future episodes.

### Use of Multiple Podcast Platforms and Social Media Engines for Promotion

To enhance use, we utilized several platforms and launched simultaneously on all of them to allow for listeners to find the episodes easily. Baby Doctor Mama podcasts are found on Apple iTunes, Spotify, Stitcher, iheartradio, and Soundcloud. Promotion of the podcast was done almost exclusively on Web-based social media platforms such as Instagram, Twitter, and Facebook, all under the name Baby Doctor Mamas. Posting on these platforms occurred before launch, and the accounts were all public facing to allow for feedback and discovery. The only other promotional move made was to launch the podcast during a national conference for pediatrics. Flyers were printed and distributed at this conference to gain listenership.

### Thoughts on Expansion and Audience Reach

**Figure 1:**
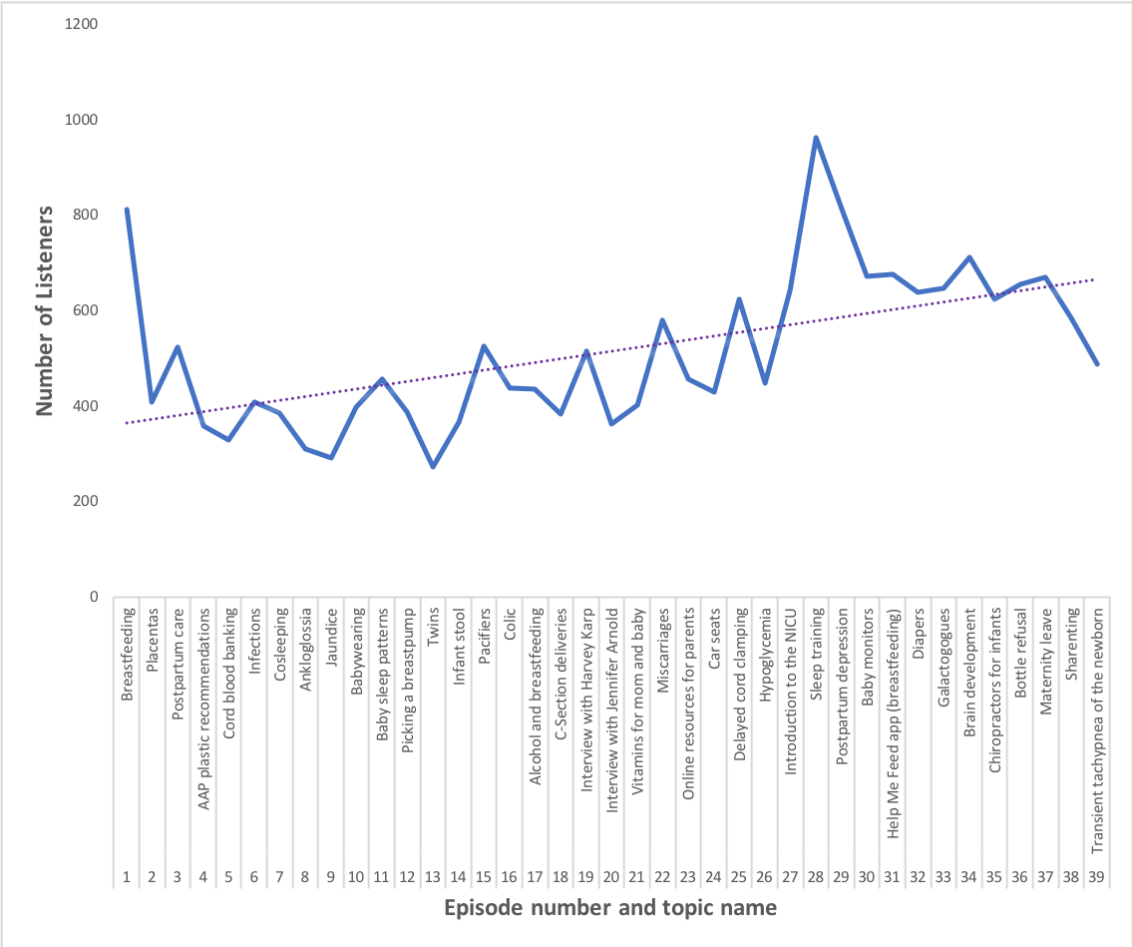
The number of listeners per episode of the Baby Doctor Mamas podcast from October 29, 2018, to September 1, 2019. The graph shows an increase in listeners over time and reviews the topics discussed in each episode. The blue solid line represents the number of listeners for each topic over time. The purple dotted line represents the mean number of listeners over time for all episodes. AAP: American Academy of Pediatrics; NICU: Neonatal Intensive Care Unit.

Initial episodes of the podcast engaged 200 to 400 listeners, and this has steadily grown to anywhere from 450 to more than 800 listeners per episode, with a maximum of 958 listeners on a sleep training episode (see Figure 1).

In less than 1 year, we have 719 followers on Instagram, 494 on Facebook, and 97 on Twitter. Tactics that have worked to increase social media following and downloads of the podcast/listens per episode include the following: having an expert or celebrity on infant topics (ie, Dr Harvey Karp or Dr Jennifer Arnold), interviewing an Instagram influencer with tens of thousands of followers and having them post on social media regarding the episode (ie, Dr Danielle Jones aka Mama Doctor Jones @mamadoctorjones with 55.5K followers on Instagram), and linking in an expert with a high twitter following and ability to promote through a department within the hospital (ie, Dr Ariel Williamson on sleep training who posted about the episode through the Health Policy Lab at the Children’s Hospital of Philadelphia).

### Conclusions

As medical podcasters and mothers, our goals are to disseminate evidence-based medicine and provide entertaining and accessible content to listeners. Although there has been steady growth of the Baby Doctor Mama podcast through social media promotion, there is still room to grow. Eventually, working in conjunction with an academic university for podcast promotion and listenership may be a key strategy for the growth and expansion of listenership to more patients/parents. Marketing the podcast differently with a focus on creating a media packet is also an option. More research is needed into how patients/parents are utilizing podcasts for medical information.
